# The Tablets, Ring, Injections as Options (TRIO) study: what young African women chose and used for future HIV and pregnancy prevention

**DOI:** 10.1002/jia2.25094

**Published:** 2018-03-30

**Authors:** Ariane van der Straten, Kawango Agot, Khatija Ahmed, Rachel Weinrib, Erica N Browne, Kgahlisho Manenzhe, Fredrick Owino, Jill Schwartz, Alexandra Minnis, on behalf of the TRIO Study Team

**Affiliations:** ^1^ Women's Global Health Imperative (WGHI) RTI International San Francisco CA USA; ^2^ Department of Medicine Center for AIDS prevention studies UCSF San Francisco CA USA; ^3^ Impact Research and Development Organization Kisumu Kenya; ^4^ Setshaba Research Centre Soshanguve South Africa; ^5^ CONRAD/EVMS Arlington VA; ^6^ School of Public Health UC Berkeley CA USA

**Keywords:** multipurpose prevention technologies, end‐user research, HIV prevention, product preference, contraception, Africa

## Abstract

**Introduction:**

Preventing HIV and unintended pregnancies are key global health priorities. To inform product rollout and to understand attributes of future multipurpose prevention technologies (MPT) associated with preference and use, we evaluated three placebo delivery forms: daily oral tablets, a monthly vaginal ring, and two monthly intramuscular injections in TRIO, a five‐month study among young Kenyan and South African women.

**Methods:**

HIV‐negative, sexually active, non‐pregnant women aged 18 to 30 were enrolled and randomized to use each placebo delivery form for one month (stage 1). Then, participants chose one product to use for two additional months (stage 2). We assessed safety, product ranking, choice, and use. We examined demographic and behavioural correlates of choice and, reciprocally, unwillingness to use in the future with logistic regression models.

**Results:**

277 women enrolled, 249 completed stage 1 and 246 completed stage 2. Median age was 23 years, 49% were Kenyan and 51% were South African. Three participants became pregnant during the study and one participant HIV‐seroconverted. There were 18 product‐related adverse events, six tablets‐related, 11 ring‐related, and one injection‐related. After trying each product, 85% preferred a TRIO product over condoms. Injections were chosen most (64%, 95% confidence interval (CI) 58%, 70%; *p *< 0.001), and by more South Africans than Kenyans (odds ratio (OR) 2.01, 95% CI: 1.17, 3.43; *p* = 0.01). There was no significant difference in choosing tablets versus ring (21%, 95% CI: 16%, 26% vs. 15%, 95% CI: 11%, 20%; *p* = 0.11). Tablet and ring adherence, based on direct observations and self‐reports, improved over time. However, participants’ self‐reported use of tablets did not match objective data from the electronic dose monitoring device. Participants were fully compliant with injections.

**Conclusion:**

In this population at risk for HIV and pregnancy, all participants agreed to choose and use a placebo MPT delivery form. A majority of participants preferred TRIO products to male condoms, an existing MPT. Injections were most liked and best used, however, they are years away from reaching the clinics. In the meantime, expanding the availability of tablets and giving access to rings can begin to fulfill the promise of choice for HIV prevention technologies and inform the development of suitable delivery forms as MPT.

## 1 Introduction

Sexually active women at risk for HIV also need to prevent unintended pregnancies. In sub‐Saharan Africa, 40% to 60% of pregnancies remain unintended 1, 2; likewise, young women account for approximately 25% of new HIV infections 3. Despite important milestones with new prevention technologies, including oral pre‐exposure prophylaxis (PrEP) and microbicides, achieving adequate use to confer protection is challenging, particularly among youth 4, 5, 6. Evidence has accumulated over the past decades that contraceptive choice increases uptake and adherence for individuals, and coverage at the population level 7, 8, a lesson that could be applied to HIV prevention strategies.

For young African women, the dual reproductive health concerns of HIV and unintended pregnancy call for dual‐purpose solutions. Multipurpose prevention technologies (MPTs) are biomedical interventions with more than one indication, such as prevention of HIV and unintended pregnancy‐ preferably in one formulation 9. An MPT that combines HIV and pregnancy prevention could offer several advantages over two single‐indication products: it may improve motivation to use and acceptability by bundling a more common outcome (pregnancy) with a rarer and more stigmatized one (HIV), it simplifies use with potential adherence optimization, and it may also decrease burden on local health systems 10, 11. Research so far has shown that African women overwhelmingly favor an MPT 12, 13, 14. However, well‐known challenges associated with condom, an existing MPT, (e.g. controlled by men, interference with sex, on‐demand use) have shown the importance of focusing on key product attributes to enhance women's ability to use 15. Importantly, few data are available on preference and choice in the context of actual product use 16, 17, 18, 19, a gap that this study directly addressed.

The TRIO (tablets, ring, injections as options) study examined acceptability, preference, choice and use of three potential MPTs among African women. Oral tablets, intra‐muscular injections and vaginal rings were selected for evaluation as they capitalize on effective contraceptives and reflect products currently pursued for HIV prevention and as MPTs 20, 21, 22. Here we sought to answer the following questions: first, what were women's preferences for the TRIO products compared to each other and to the male condom, a known and available MPT? Second, which of the TRIO products was most often chosen, and reciprocally, which product(s) were women unwilling to use in the future? Third, how well were women able to use the products they had chosen?

## 2 Methods

TRIO included a five‐month prospective randomized, cross‐over clinical study conducted between December 2015 and December 2016 at two African sites: Impact Research and Development Organization (IRDO) in Kisumu, Kenya and Setshaba Research Centre (SRC) in Soshanguve, South Africa. Written informed consent was obtained from all participants prior to any procedures. Ethical approval for the study was obtained from Pharma‐Ethics (South Africa) and KEMRI Scientific and Ethics Review Unit (Kenya).

**Figure 1:**
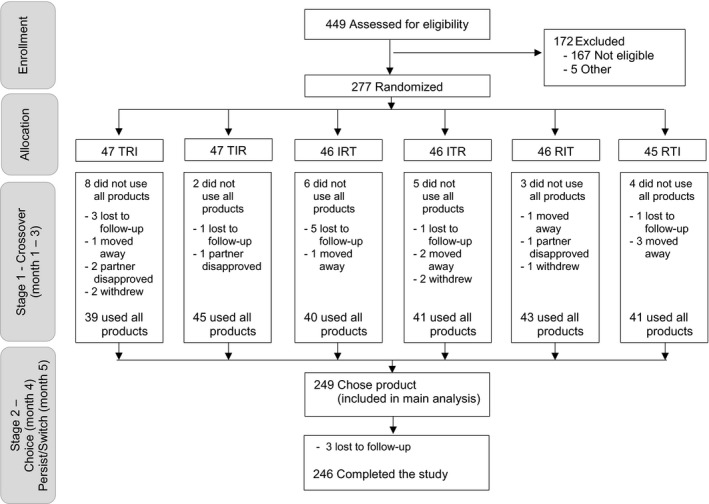
TRIO study flow chart. Product Key: I, Injectable; R, Ring; T, Tablet. TRIO study design, participants disposition and analytical sample. At enrolment into stage 1, participants were randomized to one of six product‐use sequences. Randomization was stratified by site and included blocks of size six to equally distribute the six treatment sequences.

The clinical study consisted of two stages: a three‐month cross‐over period in which women used each product for one month (stage 1), followed by choice of a product to use for another two months (stage 2). We recruited from peri‐urban communities near study clinics; detailed eligibility, recruitment and enrolment procedures are described elsewhere 23. Eligible participants were HIV‐negative, non‐pregnant women 18 to 30 years old who were not wanting to get pregnant in the next six months and had not participated in any prior HIV‐prevention or MPT studies. At enrolment, participants were randomized to one of six product‐use sequences (Figure 1).

### 2.1 Study products

In TRIO, only placebo products were used, as the goal was to investigate the three delivery forms, uncoupled from active ingredients’ side effects or effectiveness. At each visit, participants received one of the following: (a) a Wisepill electronic dose monitoring device (Wisepill Technologies, Cape Town, South Africa) with 30 placebo Truvada tablets (representing a co‐formulated tablet; Gilead Sciences, Foster City, CA) for daily oral dosing during one month; (b) a silicone elastomer placebo vaginal ring 24, 25 (International Partnership for Microbicides, Silver Spring, MD) or (c) two 2 mL saline injections administered at the clinic, one in each gluteal muscle (as used in the HPTN‐076 trial 26).

Male condoms were distributed at every visit to all participants with demonstration and risk reduction counselling. When answering surveys, participants were asked to assume that all the products were as effective as condoms in preventing HIV and pregnancy. Participants could use any contraceptive method of their choice during the study.

### 2.2 Procedures

At baseline, participants watched a brief animated educational video explaining how to use each product, including dosing frequency, and received their first product with instructions for use. The site pharmacist dispensed tablets and observed the first dosing. Ring insertion into the vagina occurred at the clinic with guided instruction by the study clinician and confirmation of correct placement. Injections were administered by study clinicians.

Participants answered questions about choice and preferences among products at entry into stages 1 and 2. Stage 2 was a two‐month use period with participants’ chosen product. After one month (month‐4 visit), participants were offered the option to switch to a different TRIO product for month‐5, the final use month. Questions related to product switching and comparisons across products were asked at months 4 and 5 respectively.

### 2.3 Measures

#### 2.3.1 Product ranking

At month‐3, participants ranked the TRIO products and condoms.

#### 2.3.2 Choice

At month‐3, each participant was asked to choose one product for stage 2 and to explain the reason for their choice. Participants also selected which product(s) they would definitely not use in the future.

#### 2.3.3 Persistence

We assessed whether the participant used her chosen product throughout stage 2 or switched to a different product.

#### 2.3.4 Adherence

We created a multicomponent binary measure based on observed product use at the visits and self‐reported use. Observed use was based on if (a) participants took their first tablet or inserted the ring while at the clinic (observed ingestion/insertion) and (b) completed monthly use at the clinic (observed final tablet ingestion or examined for ring placement). For self‐reported use, participants were asked which weeks of the previous month the product was used. They were also asked whether they took tablets “every day or almost every day” or, whether the ring came out on its own or was ever removed during the month. Adherence for tablets was defined by meeting all of the following: initiation and completion of oral regimen in the clinic, reported tablet use “every day or almost every day,” and reported use during each week of the month. For electronic monitoring, a participant was considered adherent based on Wisepill data if the container was opened on at least 80% of the days during the month. Adherence for the ring was defined by all of the following: ring inserted at the initial visit, ring in place when returned, reported use during each week of the month, and ring reported to never have been removed or come out during the month.

#### 2.3.5 Risk score

Seven behavioural measures were combined to create a behavioural risk score (range 0 to 7). One point was assigned for each of the following: knows or suspects partner has other partners, no contraceptive use and sexually active, did not use condom during last sex act, and, in the past 30 days used drugs, binge drank alcohol, had >1 sexual partner, or any transactional sex.

### 2.4 Laboratory tests and safety

At baseline and final visits, we conducted testing for pregnancy (QuickVue One‐Step HCG Urine test) and HIV (Alere Determine HIV 1/2; with the following confirmatory tests: Premier First Response HIV 1‐2.0 Card at IRDO and Trinity Biotech Uni‐Gold Recombigen HIV 1&2 at SRC).

Participants received a pelvic examination and clinical assessment as part of screening procedures to rule out current urogenital conditions; treatment and referrals for possible STIs were provided per local syndromic management guidelines. Safety was monitored throughout the study to assess and compare adverse events and social harms associated with each delivery form (independently from any drug side effects). At every follow‐up, participants met with a study clinician to review their health, medication and contraceptive use, and any product‐specific reactions or complaints since the last study visit. Adverse events (AEs) were reported by the clinician using a standard form, summarized and reviewed monthly by an independent medical monitor to confirm determination of relatedness to the study product. During stage 1, study staff made check‐in phone calls midway through each follow‐up month to collect any problems with participants’ current product.

### 2.5 Sample size and analysis

Assuming the proportion who chose each product would be ≤0.40, a final sample of 250 was estimated to provide 80% power to detect a difference in proportion of 0.15, with a two‐sided alpha of 0.05.

Our analysis focused on preference and choice outcomes during stage 2 and use and safety data for the entire five‐month clinical study. We examined bivariate associations between sociodemographic and behavioural covariates and the outcomes of product choice and disinterest in future use using Fisher's exact tests (categorical) and t‐tests (continuous). We used logistic regression models to assess associations between each background characteristic and product (a) choice and (b) disinterest in future use, controlling for randomization sequence, country, and age. Exploratory analyses of differences in adherence per alcohol use and age < 22 were assessed, using logistic regression models, controlling for randomization sequence and country. All analyses were conducted, using Stata version 14.0 (StataCorp, College Station, TX).

## 3 Results

Overall, 277 women were randomized into stage 1, 140 in Soshanguve, South Africa and 137 in Kisumu, Kenya; 249 (90%) completed stage 1 and 246 (89%) completed stage 2 (Figure 1). Product sequence was not associated with retention (*p* = 0.70). Participants’ median age was 23 years, 94% currently had a primary partner, and 78% were parous (Table 1). Participants in Kenya and South Africa differed on many evaluated characteristics, including marriage or cohabitation (48% versus 9%), source of income (50% versus 13%), food insecurity (70% versus 39%), and history of transactional sex (18% versus 6%; all *p* ≤ 0.001). Overall, 92% of participants had ever used a male condom for family planning and/or HIV prevention, 70% had ever used an injectable, and 26% had ever used oral contraceptives. South African participants were more likely to have ever used an injectable whereas Kenyan participants were more likely to have ever used contraceptive implants. At enrolment, more South African women were currently, using injectables for contraception (53% versus 30%, *p* < 0.001). Overall, 11% were currently not using any contraception.

**Table 1:** Demographic characteristics of study population

	Soshanguve, RSA	Kisumu, Kenya	Total	
	N (%) 140 (100)	N (%) 137 (100)	N (%) 277 (100)	*p*‐value
Age				0.99
Median (IQR)	23 (21 to 26)	23 (21 to 26)	23 (21 to 26)	
18 to 24	92 (66)	91 (66)	183 (66)	
25 to 30	48 (34)	46 (34)	94 (34)	
Currently have a primary partner	135 (96)	126 (92)	261 (94)	0.13
Married or cohabiting	13 (9)	66 (48)	79 (29)	<0.001
Currently have a casual sex partner	19 (14)	31 (23)	50 (18)	0.06
Exchange sex ever	8 (6)	25 (18)	33 (12)	0.001
Parity >0	109 (78)	107 (78)	216 (78)	0.99
Completed secondary school	86 (61)	57 (42)	143 (52)	0.001
Earns an income	18 (13)	68 (50)	86 (31)	<0.001
Food insecurity past 4 weeks				<0.001
Never	86 (61)	42 (31)	128 (46)	
Rarely or sometimes	36 (26)	72 (53)	108 (39)	
Often	18 (13)	23 (17)	41 (15)	
How often attend religious services each week				<0.001
Sometimes/often	117 (84)	135 (98)	252 (91)	
Never/no religion	23 (16)	2 (2)	25 (9)	
Any alcohol use past 4 weeks	62 (49)	25 (20)	87 (35)	<0.001
Worried contract HIV in next 12 months				0.45
Not at all/a little	87 (62)	92 (67)	179 (65)	
Somewhat/very/extremely	53 (38)	45 (33)	98 (35)	
Ever diagnosed with STI	13 (9)	2 (2)	15 (5)	0.003
Behavioural risk score, mean (SD)^a^	1.4 (1.2)	1.4 (1.0)	1.4 (1.1)	0.55
Has privacy in the home	128 (91)	100 (73)	228 (82)	<0.001
Methods ever used^b^				
Male condom	131 (94)	124 (91)	255 (92)	0.38
Injectable	113 (81)	81 (59)	194 (70)	<0.001
Implants	36 (26)	62 (45)	98 (35)	0.001
Pills	33 (24)	39 (29)	72 (26)	0.41
IUD	7 (5)	7 (5)	14 (5)	0.99
Female condom	7 (5)	18 (13)	25 (9)	0.02
Other	3 (2)	7 (5)	10 (4)	0.21
None	1 (1)	1 (1)	2 (1)	0.99
Methods used at enrolment^b^				
Male condom	80 (57)	61 (45)	141 (51)	0.04
Injectable	74 (53)	41 (30)	115 (42)	<0.001
Implants	30 (21)	37 (27)	67 (24)	0.33
Pills	9 (6)	9 (7)	18 (7)	0.99
IUD	7 (5)	4 (3)	11 (4)	0.54
Female condom	2 (1)	6 (4)	8 (3)	0.17
Other	2 (1)	4 (3)	6 (2)	0.44
None	12 (9)	17 (12)	29 (11)	0.33

IQR, interquartile range; RSA, Republic of South Africa

a Composite of 7 measures (range 0 to 7).

b Can select more than one.

### 3.1 Product ranking and preference

**Figure 2:**
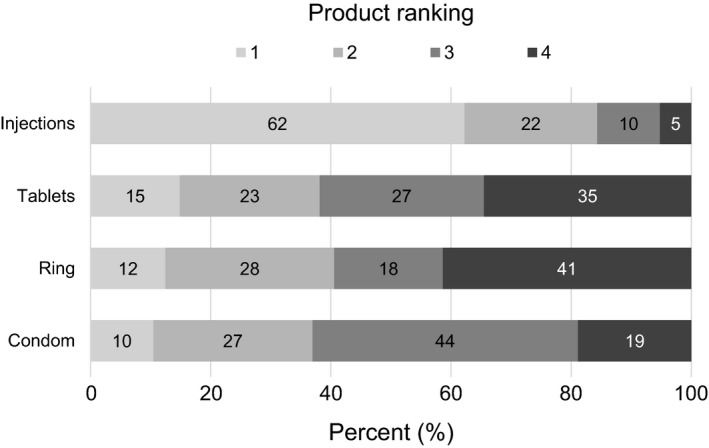
Ranking of TRIO study products with condoms for both HIV and pregnancy prevention. After trying each study product for one month, participants (n = 249) were asked to rank the products and condoms in order of preference, assuming all products had the same effectiveness as male condoms to prevent HIV and unplanned pregnancy.

After using each TRIO product for one month in stage 1, 211 participants (85%) stated they would prefer one of the study products over condoms for HIV and pregnancy prevention. When asked to rank the TRIO products with condoms in order of preference, 155 women (62%, 95% CI: 56%, 68%) ranked injections first, 37 (15%, 95% CI: 11%, 20%) ranked tablets first, 31 (12%, 95% CI: 9%, 17%) ranked ring first, and 26 (10%, 95% CI: 7%, 15%) ranked condoms first. The ring (41%) and tablets (35%) were most likely to be ranked least preferred (Figure 2).

### 3.2 Product choice and persistence

All 249 participants chose a TRIO product to use for stage 2. Choice aligned closely with stated preference; 64% chose injections (95% CI: 58%, 70%), 21% chose tablets (95% CI: 16%, 26%), and 15% chose the ring (95% CI: 11%, 20%; Table 2). Significantly more participants chose injections (*p* < 0.001), but there was no significant difference between those choosing tablets versus ring (*p* = 0.11). Overall, 17% of participants explained that their choice of product was out of convenience. Other frequent explanations for choosing the tablets was lack of side effects (14%); for the ring it was comfort (24%) followed by dosing frequency (11%) and peace of mind (11%); for the injections it was dosing frequency (26%) and peace of mind (24%), defined as not having to worry about forgetting. Twenty‐three participants (9%) chose to use a product that was not their most preferred one.

**Table 2:** Participants’ behaviour and assessments of TRIO study products (N = 249)

	TRIO study product		
	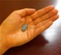	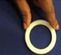	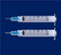
	Tablets	Ring	Injections
	N (%)	N (%)	N (%)
At month 3			
Ranked as top preferred MPT^a^	37 (15)	31 (12)	155 (62)
Ranked as least preferred MPT^a^	86 (35)	103 (41)	13 (5)
Chose to use product in stage 2^b^	52 (21)	37 (15)	160 (64)
Disinterest in using product in the future^c^	108 (43)	118 (47)	13 (5)
At month 4			
Switched to product	22 (9)	12 (5)	16 (6)
Switched away from product	11 (4)	12 (5)	27 (11)

MPT, multipurpose prevention technology.

a Ranking (from 1 to 4) was among the three TRIO products and male condoms, a known MPT, assuming all products had the same effectiveness as male condoms to prevent HIV and unplanned pregnancy.

b Among those, twenty‐three participants (9%) chose to use a product that was not their most preferred one. Of those 23, three‐quarters (n = 17) preferred injections but chose to use tablets (n = 10) or the ring (n = 7). Three of the 23 participants chose a product they would “definitely not consider using in the future:” two chose tablets (reason provided was they “are easy” and “not painful”), and one chose the ring (because she initially found it uncomfortable but wanted to try it again).

c Participants were presented with the 3 TRIO products and could choose more than one option. There were 16 participants who openly indicated they would consider using all three products in the future.

When asked which TRIO product(s) participants would definitely not consider using in the future, this nearly evenly segmented the sample: 43% indicated tablets, 47% indicated ring, with only six women (2%) reporting disinterest in both the ring and tablets. Five percent were disinterested in injections. Sixteen women did not select a product because they would consider using all products in the future (Table 2).

There were 50 participants (20%) who switched products at month 4 (Table 2). There was no association between switching and initial product choice (*p* = 0.11). Of those who switched, 44% switched to tablets, 24% to the ring and 32% to injections. Therefore, at month 4, 60% chose injections (95% CI: 53%, 66%), 26% chose tablets (95% CI: 20%, 31%) and 15% chose the ring (95% CI: 11%, 20%).

### 3.3 Factors associated with product choice

South African women had a twofold increased odds of choosing injections (versus not), compared with Kenyan women (adjusted odds ratio (AOR) 2.01, 95% confidence interval (CI): 1.17, 3.43; *p* = 0.01), and Kenyan women had similar odds of choosing tablets (AOR 2.11, 95% CI: 1.11, 3.99; *p* = 0.02). There was no significant difference in choice of the ring by country (AOR 1.33, 95% CI: 0.65, 2.72; *p* = 0.43). Choice was not significantly associated with any other background characteristics, including prior and current contraceptive methods (all *p* > 0.05, Table S1). However, when examining factors associated with unwillingness to use a product in the future, women with their own source of income, who were nulliparous, who did not have a casual sex partner, and who did not have a private place in their home had a twofold increase in the odds of being disinterested in the ring (all *p* < 0.05, Table 3). In contrast, having a private place in the home was associated with increased odds of disinterest in tablets. All modelling results can be found in Table S2.

**Table 3:** Participant demographics and baseline characteristics associated with product choice at month 3 and unwillingness to use the product in the future. (N = 249)

	Choice		Disinterest/unwilling to use in the future	
TRIO Product	Adjusted OR (95% CI)	*p*‐value	Adjusted OR (95% CI)	*p*‐value
Tablets				
Has privacy in the home	0.47 (0.22, 1.01)	0.05	2.36 (1.08, 5.14)	0.03
Ring				
Have own source of income	0.53 (0.23, 1.24)	0.14	2.05 (1.09, 3.85)	0.03
Does not have a casual sex partner	0.55 (0.24, 1.23)	0.14	2.01 (1.02, 3.94)	0.04
Nulliparous	0.99 (0.40, 2.49)	0.99	1.99 (1.03, 3.86)	0.04
Has privacy in the home	2.29 (0.73, 7.21)	0.16	0.38 (0.18, 0.80)	0.01

No characteristics were significantly associated (*p* < 0.05) with choice or disinterest in injections. Here, *p*‐values from logistic regression models adjusted for country, randomization sequence, and age. CI, confidence interval.

### 3.4 Adherence and electronic monitoring

Adherence (per the composite measure) during stage 1 was better among those who chose to use that product for Stage 2 compared to those who chose a different product, although the differences were not statistically significant (*p* ≥ 0.05). Forty‐nine percent (95% CI: 33%, 65%) of those who chose the ring were adherent in stage 1 compared to 40% who did not choose the ring (95% CI: 34%, 47%); 62% of those who chose tablets were adherent in stage 1 (95% CI: 47%, 74%) compared to 44% who did not choose tablets (95% CI: 37%, 51%). Notably, tablet use based on Wisepill data did not suggest any difference in stage 1 between tablet choosers (30%) and non‐choosers (31%). Injection adherence was documented for those receiving the injection at the clinic following randomization to this product or choice of injections during stage 2. No participants declined the injection in stage 1 or following choice of this product in stage 2.

**Figure 3:**
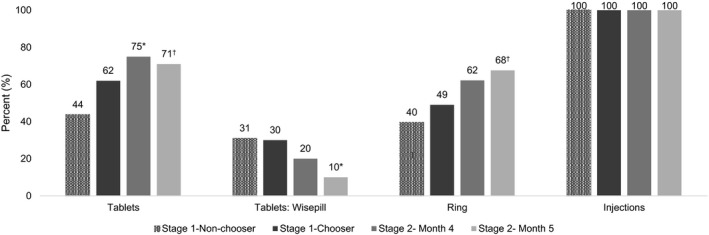
Product adherence during the TRIO study. Adherence was a multicomponent measure based on self‐reported use and direct observation of use during the study visits (initiation and return visit after 1 month of use). For injections, adherence was based on receiving two injections at the initiation visit by the study clinician. Adherence during stage 1 is shown for those who chose to use the product in stage 2 compared to those who did not chose to use the product in stage 2. Adherence improved during stage 2 for tablets (*p* = 0.04) and ring (*p* = 0.06). For tablets, objective use data was also available from Wisepill containers, which electronically track the opening of the tablet container (second set of bars from the left). Participants were considered adherent per Wisepill if the container was opened at least once per day for 80% of days during the month. For tablet use per Wisepill, there was a significant decrease in use over time (*p* = 0.002). *p < 0.05 †*p* < 0.10; *p*‐values from mixed‐effect logistic regression model.

Among those who chose tablets during month 4, 96% took their first tablet at the clinic, 94% took their final tablet at the return visit, and 75% were adherent (per the composite measure, 95% CI: 61%, 85%). Participants’ adherence for the tablets did not match data from the Wisepill container. The proportion of participants who opened the Wisepill at least once per day for 80% of the days in the month significantly decreased from 30% in stage 1 to 20% in month 4 and 10% in month 5 (*p* = 0.002). Among those who chose to use the ring during month 4, 100% had the ring inserted at the clinic, 81% had the ring in place at the return visit, and 62% were overall adherent (95% CI: 45%, 77%; Figure 3).

In stage 1, odds of adherence for the ring and tablets were significantly lower among those who indicated any alcohol use in the past 4 weeks prior to enrolment: ring adherence AOR 0.48, 95% CI: 0.29, 0.79; *p* = 0.004 and tablets adherence AOR 0.51, 95% CI: 0.28, 0.92; *p* = 0.03. Odds of adherence while using the ring were significantly higher for women >21 years of age (AOR 1.88, 95% CI: 1.10, 3.22; *p* = 0.02). There was no significant difference in adherence for tablets by age (per adherence measure or electronic monitoring).

### 3.5 Safety of placebo delivery forms

There were 18 TRIO product‐related adverse events (AEs): six tablets‐related, 11 ring‐related, and one related to injections. Fifteen of 18 (83%) occurred during stage 1 and most were considered mild (15 mild and 3 moderate). AEs while using the ring included vaginal pruritis (n = 5), lower abdominal pain (n = 4), discomfort (n = 1) and pelvic inflammatory disease (n = 1). Five (45%) discontinued use, all during stage 1. AEs while using tablets were nausea (n = 2), vomiting (n = 2), lower abdominal pain (n = 1) and body rash (n = 1). Two (33%) discontinued use in stage 1. One participant reported an injection site reaction that lasted >24 hours. Four participants reported study‐related social harms, three while using the ring (one experienced stigma and two reported their partner was angry and demanded the ring to be removed) and one while using tablets (conflict with participant's parents who thought the tablets were antiretroviral therapy).

There were 705 mid‐visit check‐in phone calls during stage 1; problems while using current study products were reported during 59 calls (8%). Half met criteria for AEs and were also reported as such. Twenty‐eight women reported problems while using tablets (most commonly reported: difficulty remembering to take them, fatigue/not feeling well, and vomiting). Twenty‐five reported problems while using the ring (most commonly reported: discomfort and partner dissatisfaction). Six women reported problems while using the injections, none were injection site reactions.

Three participants became pregnant (one in South Africa and two in Kenya) and one participant in Kenya tested positive for HIV at the end of the study.

## 4 Discussion

In this study conducted among young African women at dual risk for HIV and unplanned pregnancy, all participants agreed to choose and use a placebo MPT delivery form. Most participants preferred a TRIO product over condoms, and all three delivery forms were safe in this study. Injections were most highly ranked, most often chosen and used with full compliance. Importantly, preference, choice and use were very similar between tablets and ring, despite rings being a completely novel delivery form for this population. Product choice was associated with country, but not with any underlying demographic characteristics evaluated. Unwillingness to use either tablets or ring in the future, a reciprocal of choice, almost equally divided the study population. Thus, to complement oral PrEP, regulatory approval and rollout of the ring can increase women's options in the near‐term by offering an alternate method for those unwilling or unable to use tablets and thus, could enhance population coverage for HIV prevention.

We previously reported that participants liked all three products more after one month of use, with the largest improvement for the ring 27. Importantly, participants were also willing to choose a new product, the ring, once they had a chance to try it. Product preference and choice were highly correlated at month 3 and indicated greatest interest for the injections, a provider‐administered, long‐acting product. This is aligned with findings from hypothetical studies, reporting that long‐acting methods, and injectables in particular, are often preferred over daily or event‐driven methods 12, 19, 28, 29. Likewise, studies of contraceptive choice suggest many women prefer long‐acting methods and opt for these methods when given comprehensive counselling and financial barriers to uptake are removed 18. To date, preference for systemic products versus topical appears to be driven in part by a greater familiarity with the systemic route of administration (e.g. oral pills, injections) combined with less concern for interference with sex 28, 29, 30. Product familiarity may also explain why so few AEs were reported with injections. Given its novelty as a product and route of administration, it is notable that the ring fared as well if not better than condoms (a known MPT) and fared similarly to oral tablets (a more familiar route of administration).

Apart from country, there were no behavioural and demographic correlates of product choice. There was important heterogeneity across sites, and low power to discern specific underlying group differences informing choice. Also, perhaps personal preference for specific product attributes was driving choice more than group characteristics. Indeed, choice was correlated with acceptability of product attributes (e.g. how the product looked, ease of using, and how much it interfered with normal activities), similarly to the attributes associated with rating a product highly in stage 1 27. At baseline, stated preference was associated with demographics and familiarity with known contraceptive delivery form 23. Here, after participants had an opportunity to try each product, contraceptive history no longer was associated with preference or choice.

Unwillingness to use tablets or ring in the future evenly segmented the sample, suggesting that adding ring to the HIV prevention method mix could offer an important alternative to oral PrEP among young women. However, stated disinterest in one product does not necessarily indicate perfect adoption of the other. Privacy in the home, also associated with higher ratings for the ring 27, seemed the main factor differentiating disinterest for the pill versus the ring. One possible interpretation is that a private room may be necessary to be able to insert or remove the ring, whereas swallowing a pill can be done anywhere. Greater disinterest for the ring among nulliparous women may be associated with a concern that the ring may impact fertility or stretch the vagina, worries expressed previously by young ring users in a clinical trial 31, 32.

Study design feasibility was demonstrated with every TRIO participant willing to try, choose and use the products. This may in part be a benefit of using placebos, with no fear of toxicity or drug‐related AE. This design is now being replicated with active tablets and ring in an open‐label clinical trial 33. Nevertheless, TRIO suffered from several limitations: our sample was quite experienced with using contraceptive methods; injections in TRIO did not fully reflect active product experiences, because the placebo injections are less painful than the active product 34, and the study did not include lead‐in and lead‐out oral dosing, which is currently part of the PrEP injections regimen 34. In other words, TRIO injections presented an optimistic scenario. Adherence measures were not comparable across the three products due to differences inherent in the products themselves. Injection administration was 100% ascertained. For the tablets, Wisepill indicated much lower use than the adherence measure. Pills may have been chosen more often by participants not interested in taking them, because they are easy not to use. We could not ascertain ring use during each month with an objective marker. Given that, we cannot determine objectively if ring monthly adherence was different from tablet use by Wisepill. Nevertheless, based on clinic observation and self‐reports, tablet and rings appeared to have similar levels of adherence, with lower completion for the ring, and lower (self‐reported) execution for the tablets. In the cross‐over period, adherence for the tablets and ring was higher amongst those who abstained from alcohol drinking and among choosers (vs. non‐choosers), suggesting that preference may help adherence, although this was not significant in this small sample. As previously reported 25, ring adherence was also higher in women older than 21 years old.

## 5 Conclusion

We examined preferences, actual choice, and use with diverse placebo delivery forms to address gaps in understanding key product attributes that influence interest in future MPT products. The research assessed preferences among injections, rings, and tablets and how preferences influenced use, to inform both product development and prevention planning. Although, injections were most liked and best used, they are still far away from reaching the clinics. In the meantime, expanding the availability of tablets and rings can start to fulfill the promise of choice in HIV prevention technologies for women and inform the development of suitable delivery forms as MPT.

## Competing interests

The authors have no competing interests to declare.

## Authors’ contributions

AvdS is the principal investigator and contributed to the study's design, implementation, analysis and led the writing of this manuscript. KA and KA are the site principal investigators and led the implementation and data collection. RW is the study coordinator and contributed to the study's design and implementation and to writing of this manuscript. EB is the study statistician and contributed to data management, analysis and writing of this manuscript. KM and FO are the site study coordinators and contributed to study implementation and data collection. JS is the medical monitor and contributed to clinical aspects of the study. AM is the co‐principal investigator and contributed to the study's design and implementation. All authors have reviewed and contributed to the writing, and have read and approve the final manuscript.

## Supporting information


**Table S1.** Logistic regression models assessing the association between each participant demographic or baseline characteristic and product choice at month 3 in the TRIO study

**Table S2.** Logistic regression models assessing the association between each participant demographic or baseline characteristic and unwillingness to use the product in the future; results from the TRIO study (December 2015 to December 2016)
