# Supplementary material for: The Tablets, Ring, Injections as Options (TRIO) study: what young African women chose and used for future HIV and pregnancy prevention
Source: J Int AIDS Soc. 2018 Mar 30;21(3):e25094. doi: 10.1002/jia2.25094 (PMC5876496; doi:10.1002/jia2.25094)
Supplement: Supplementary file 1 — Table S1. Logistic regression models assessing the association between each participant demographic or baseline characteristic and product choice at month 3 in the TRIO study [file JIA2-21-e25094-s001.docx]

Supplemental Table 1. Logistic regression models assessing the association between each participant demographic or baseline characteristic and product choice at month 3 in the TRIO study.

|  | Chose Tablets  (N=249) | | | Chose Ring (N=249) | | | Chose Injections (N=249) | | |
| --- | --- | --- | --- | --- | --- | --- | --- | --- | --- |
|  | AOR | 95% CI | p-value | AOR | 95% CI | p-value | AOR | 95% CI | p-value |
| Kenya (vs South Africa) | 2.10* | (1.11 - 3.99) | 0.02 | 1.33 | (0.65 - 2.73) | 0.43 | 0.50* | (0.29 - 0.85) | 0.01 |
| Age group, years  (25-30 vs 18-24) | 1.13 | (0.59 - 2.21) | 0.70 | 1.12 | (0.84 - 1.51) | 0.45 | 0.84 | (0.48 - 1.49) | 0.55 |
| Currently have a primary partner | 1.17 | (0.30 - 4.56) | 0.83 | 0.38 | (0.11 - 1.37) | 0.14 | 1.68 | (0.55 - 5.08) | 0.36 |
| Married or cohabiting | 1.28 | (0.61 - 2.69) | 0.51 | 0.70 | (0.28 - 1.73) | 0.44 | 1.00 | (0.52 - 1.92) | 0.99 |
| Currently have a casual sex partner | 0.72 | (0.31 - 1.63) | 0.43 | 1.83 | (0.81 - 4.14) | 0.14 | 0.87 | (0.45 - 1.68) | 0.67 |
| Exchange sex ever | 1.80 | (0.77 - 4.18) | 0.17 | 1.39 | (0.50 - 3.83) | 0.52 | 0.52 | (0.24 - 1.13) | 0.10 |
| Parity >0 | 0.73 | (0.33 - 1.64) | 0.45 | 1.00 | (0.40 - 2.49) | 1.00 | 1.24 | (0.63 - 2.46) | 0.54 |
| Worried contract HIV | 1.55 | (0.81 - 2.95) | 0.18 | 0.72 | (0.33 - 1.55) | 0.40 | 0.87 | (0.50 - 1.51) | 0.61 |
| Completed secondary school | 0.96 | (0.50 - 1.86) | 0.91 | 0.66 | (0.31 - 1.39) | 0.27 | 1.31 | (0.75 - 2.28) | 0.35 |
| Earns an income | 0.82 | (0.39 - 1.72) | 0.60 | 1.87 | (0.81 - 4.33) | 0.14 | 0.81 | (1.43 - 1.52) | 0.51 |
| Food insecurity, past4week  Never | ref |  |  | ref |  |  | ref |  |  |
| Rarely or sometimes | 1.19 | (0.58 - 2.47) | 0.64 | 1.56 | (0.67 - 3.61) | 0.30 | 0.69 | (0.37 - 1.29) | 0.24 |
| Often | 0.98 | (0.38 - 2.47) | 0.97 | 1.02 | (0.33 - 3.18) | 0.97 | 1.01 | (0.45 - 2.29) | 0.97 |
| Attend religious services | 2.05 | (0.44 - 9.54) | 0.36 | 0.72 | (0.21 - 2.45) | 0.60 | 0.84 | (0.30 - 2.33) | 0.73 |
| Has privacy in the home | 0.47 | (0.22 - 1.01) | 0.05 | 2.29 | (0.73 - 7.21) | 0.16 | 1.26 | (0.61 - 2.58) | 0.53 |
| *Contraceptive method ever used* | | | |  |  |  |  |  |  |
| Injectable | 1.13 | (0.55 - 2.35) | 0.74 | 0.56 | (0.25 - 1.25) | 0.16 | 1.27 | (0.69 - 2.36) | 0.44 |
| Pills | 1.56 | (0.79 - 3.08) | 0.20 | 0.82 | (0.36 - 1.88) | 0.64 | 0.79 | (0.44 - 1.44) | 0.44 |
| Female condom, diaphragm, gel, or IUD | 1.00 | (0.40 - 2.46) | 0.99 | 0.69 | (0.22 - 2.19) | 0.53 | 1.21 | (0.54 - 2.70) | 0.65 |
| Implants | 0.70 | (0.35 - 1.40) | 0.32 | 1.20 | (0.56 - 2.58) | 0.63 | 1.16 | (0.65 - 2.07) | 0.61 |
| Currently using injectable contraceptive | 1.05 | (0.55 - 2.03) | 0.87 | 0.96 | (0.46 - 2.02) | 0.92 | 0.98 | (0.56 - 1.71) | 0.95 |
| Currently using hormonal birth control method | 1.00 | (0.99 - 1.02) | 0.47 | 1.00 | (0.98 - 1.02) | 0.90 | 1.00 | (0.99 - 1.01) | 0.58 |
| Currently using male condoms | 0.99 | (0.52 - 1.90) | 0.99 | 1.39 | (0.66 - 2.91) | 0.38 | 0.83 | (0.48 - 1.45) | 0.52 |
| *Most important attribute when choosing a product* | | | |  |  |  |  |  |  |
| Side effects | 0.58 | (0.26 - 1.32) | 0.20 | 0.67 | (0.26 - 1.70) | 0.40 | 1.81 | (0.91 - 3.59) | 0.09 |
| Availability/access | 1.37 | (0.63 - 2.99) | 0.43 | 0.80 | (0.30 - 2.14) | 0.66 | 0.87 | (0.43 - 1.76) | 0.71 |
| Frequency of use | 0.74 | (0.30 - 1.81) | 0.51 | 1.18 | (0.47 - 2.95) | 0.72 | 1.11 | (0.54 - 2.26) | 0.78 |
| *Study related characteristics* | | |  |  |  |  |  |  |  |
| Reported discomfort/pain while using ring | 1.99 | (0.67 - 5.93) | 0.22 | 0.94 | (0.24 - 3.60) | 0.92 | 0.60 | (0.22 - 1.64) | 0.32 |
| Reported issue with ring during check-in | 2.31 | (0.78 - 6.89) | 0.13 | 0.75 | (0.16 - 3.50) | 0.71 | 0.59 | (0.21 - 1.63) | 0.31 |
| Reported issue with tables during check-in | 0.88 | (0.30 - 2.53) | 0.81 | 0.82 | (0.23 - 2.94) | 0.76 | 1.22 | (0.49 - 3.02) | 0.67 |
| Experienced a product AE | 0.42 | (0.09 - 2.01) | 0.28 | 0.35 | (0.04 - 2.82) | 0.32 | 3.08 | (0.82 - 11.60) | 0.10 |
| * p<0.05; AOR: Adjusted odds ratio. All models were adjusted for age group, randomization sequence, and country | | | | | | | | | |
