# Supplementary material for: The Tablets, Ring, Injections as Options (TRIO) study: what young African women chose and used for future HIV and pregnancy prevention
Source: J Int AIDS Soc. 2018 Mar 30;21(3):e25094. doi: 10.1002/jia2.25094 (PMC5876496; doi:10.1002/jia2.25094)
Supplement: Supplementary file 2 — Table S2. Logistic regression models assessing the association between each participant demographic or baseline characteristic and unwillingness to use the product in the future; results from the TRIO study (December 2015 to December 2016) [file JIA2-21-e25094-s002.docx]

Supplemental Table 2. Logistic regression models assessing the association between each participant demographic or baseline characteristic and unwillingness to use the product in the future; results from the TRIO study (December 2015 - December 2016).

|  | Disinterest/Unwilling to use in the future^a^ | | | | | |
| --- | --- | --- | --- | --- | --- | --- |
|  | Tablets (N=249) | | | Ring (N=249) | | |
|  | AOR | 95% CI | p-value | AOR | 95% CI | p-value |
| Kenya (vs South Africa) | 0.47* | (0.39 - 0.58) | <0.001 | 1.66* | (1.36 - 2.04) | <0.001 |
| Age group, years (25-30 vs 18-24) | 1.16 | (0.94 - 1.44) | 0.18 | 0.98 | (0.79 - 1.21) | 0.84 |
| Currently have a primary partner | 0.69 | (0.23 - 2.07) | 0.51 | 2.16 | (0.68 - 6.88) | 0.19 |
| Married or cohabiting | 1.26 | (0.65 - 2.42) | 0.49 | 0.90 | (0.47 - 1.69) | 0.73 |
| Currently have a casual sex partner | 0.87 | (0.45 - 1.70) | 0.69 | 0.50* | (0.25 - 0.98) | 0.04 |
| Exchange sex ever | 1.30 | (0.59 - 2.88) | 0.52 | 0.72 | (0.33 - 1.59) | 0.42 |
| Parity >0 | 1.60 | (0.82 - 3.13) | 0.17 | 0.50* | (0.26 - 0.97) | 0.04 |
| Completed secondary school | 0.95 | (0.55 - 1.64) | 0.86 | 1.39 | (0.81 - 2.38) | 0.23 |
| Earns an income | 1.64 | (0.87 - 3.09) | 0.12 | 0.49* | (0.26 - 0.92) | 0.03 |
| Food insecurity past 4 weeks   Never | ref |  |  | ref |  |  |
| Rarely or sometimes | 1.00 | (0.54 - 1.83) | 1.00 | 1.13 | (0.62 - 2.06) | 0.68 |
| Often | 1.32 | (0.60 - 2.91) | 0.48 | 1.15 | (0.53 - 2.52) | 0.72 |
| Attend religious services | 1.07 | (0.42 - 2.70) | 0.89 | 0.88 | (0.35 - 2.24) | 0.80 |
| Has privacy in the home | 2.36* | (1.08 - 5.14) | 0.03 | 0.38* | (0.18 - 0.80) | 0.01 |
| Worried contract HIV | 0.58 | (0.33 - 1.01) | 0.05 | 1.14 | (0.67 - 1.95) | 0.62 |
| *Contraceptive method ever used* |  |  |  |  |  |  |
| Injectable | 0.83 | (0.45 - 1.53) | 0.55 | 1.27 | (0.70 - 2.34) | 0.43 |
| Pills | 0.55 | (0.30 - 1.01) | 0.05 | 1.38 | (0.77 - 2.46) | 0.28 |
| Female condom, diaphragm, gel, or IUD | 0.74 | (0.34 - 1.64) | 0.46 | 1.08 | (0.50 - 2.31) | 0.85 |
| Implants | 1.30 | (0.74 - 2.29) | 0.35 | 0.59* | (0.33 - 1.03) | 0.06 |
| Currently using injectable contraceptive | 1.18 | (0.69 - 2.01) | 0.54 | 0.80 | (0.47 - 1.35) | 0.40 |
| Currently using hormonal birth control method | 1.51 | (0.69 - 3.30) | 0.30 | 0.58 | (0.27 - 1.23) | 0.16 |
| *Most important attribute when choosing a product* |  |  |  |  |  |  |
| Side effects | 0.99 | (0.52 - 1.88) | 0.97 | 1.13 | (0.59 - 2.15) | 0.71 |
| Availability/access | 0.85 | (0.42 - 1.74) | 0.66 | 0.79 | (0.40 - 1.58) | 0.51 |
| Frequency of use | 1.43 | (0.74 - 2.76) | 0.29 | 0.76 | (0.39 - 1.49) | 0.43 |
| *Study related characteristics* |  |  |  |  |  |  |
| Reported discomfort/pain while using ring | 0.51 | (0.18 - 1.46) | 0.21 | 1.69 | (0.63 - 4.53) | 0.30 |
| Reported issue with ring during check-in | 0.86 | (0.32 - 2.32) | 0.76 | 1.70 | (0.63 - 4.59) | 0.29 |
| Reported issue with tables during check-in | 2.22 | (0.94 - 5.25) | 0.07 | 0.71 | (0.30 - 1.67) | 0.43 |
| Experienced a product AE | 0.58 | (0.19 - 1.83) | 0.36 | 1.58 | (0.53 - 4.69) | 0.41 |
| * p<0.05; AOR: Adjusted odds ratio. All models were adjusted for age group, randomization sequence, and country  ^a^Injections not included because only n=13 (5%) were unwilling to use injections in the future | | | | | | |
